# Real-time forecasting of an epidemic using a discrete time stochastic model: a case study of pandemic influenza (H1N1-2009)

**DOI:** 10.1186/1475-925X-10-15

**Published:** 2011-02-16

**Authors:** Hiroshi Nishiura

**Affiliations:** 1 PRESTO, Japan Science and Technology Agency, 4-1-8 Honcho, Kawaguchi, Saitama 332-0012, Japan; 2 Theoretical Epidemiology, University of Utrecht, Yalelaan 7, Utrecht, 3584 CL, The Netherlands; 3 School of Public Health, The University of Hong Kong, Pokfulam, Special Administrative Region, Hong Kong

## Abstract

**Background:**

Real-time forecasting of epidemics, especially those based on a likelihood-based approach, is understudied. This study aimed to develop a simple method that can be used for the real-time epidemic forecasting.

**Methods:**

A discrete time stochastic model, accounting for demographic stochasticity and conditional measurement, was developed and applied as a case study to the weekly incidence of pandemic influenza (H1N1-2009) in Japan. By imposing a branching process approximation and by assuming the linear growth of cases within each reporting interval, the epidemic curve is predicted using only two parameters. The uncertainty bounds of the forecasts are computed using chains of conditional offspring distributions.

**Results:**

The quality of the forecasts made before the epidemic peak appears largely to depend on obtaining valid parameter estimates. The forecasts of both weekly incidence and final epidemic size greatly improved at and after the epidemic peak with all the observed data points falling within the uncertainty bounds.

**Conclusions:**

Real-time forecasting using the discrete time stochastic model with its simple computation of the uncertainty bounds was successful. Because of the simplistic model structure, the proposed model has the potential to additionally account for various types of heterogeneity, time-dependent transmission dynamics and epidemiological details. The impact of such complexities on forecasting should be explored when the data become available as part of the disease surveillance.

## Background

Mathematical models in population biology and epidemiology have greatly progressed during the past few decades, supporting the argument for the relevance of theoretical models to the study of empirical observations [1,2]. The transmission dynamics of infectious diseases have been well studied using modeling methods, facilitating our understanding of the mechanisms of disease spread [3-5], allowing the optimization of infectious disease control, and influencing public health policymaking [4,6]. Of the various diseases that have been studied, the transmission dynamics of influenza have attracted much scientific interest, and from the beginning of the 2009 pandemic, mathematical modeling has progressed our understanding of the epidemiological dynamics of influenza (H1N1-2009) [7]. Among the various applications of mathematical models to infectious disease epidemiology, future prediction is an area that has been understudied and methods for real-time and long-term prediction in large populations have yet to be sought [8-10]. A vast amount of past quantitative modeling effort has been devoted to the inverse problem methodology which focuses on statistical estimations of model parameters and in which the process of model building imposes strong assumptions about the underlying transmission dynamics [11,12].

Prediction has two components: forecasting and projection [13]. A forecast is a quantitative attempt to predict what will happen in the future, while a projection is an attempt to describe what would happen under certain assumptions and hypotheses. Given the many studies that have examined 'what if' scenarios of an influenza pandemic using a number of plausible parameter settings [14,15], in a sense, one could regard the projection of influenza as having been widely studied. However, except for the monitoring and detection of outbreaks based on time series surveillance data [16], quantitative methods for forecasting have yet to be fully established. Although the real-time estimation of model parameters has been proposed with, for example, the aim of assessing the effectiveness of certain control measures in real-time [17], as mentioned above, such studies tended to focus on parameter estimation and quantitative forecasting has been understudied. During the course of an epidemic, it may be important to forecast the future course of the epidemic in real-time.

To date, three different approaches have been proposed for the real-time forecasting of influenza. The first employs a parsimonious, but flexible, power-law logistic equation to directly fit the parametric model (the analytical solution) to epidemic curves [18,19]. Despite the omission of the so-called "dependent happening", defined as an epidemiological phenomenon in which the risk of infection in one individual depends on the risk in other individuals in the same population unit, and the use of a simplistic minimization of the sum of squared errors, an SIR (susceptible-infected-recovered) epidemic model is known to be approximated by a family of logistic equations [20,21], and the flexible power-law logistic equation has been shown to yield reasonable fits to empirical data of H1N1-2009 [19]. A second approach employs a deterministic compartmental model to describe epidemic curves of pandemics that occurred during the 20th century [22]. This model has been shown to yield very good fits to the data, although the fitting procedure using the deterministic model requires the estimation of a total of nine parameters and computing the uncertainty bounds of forecasts is complex. One can, of course, reduce the complexity by reducing the number of unknown parameters before implementing the forecasting. The third, a hybrid stochastic epidemic model that employs a Bayesian method, was applied to H1N1-2009 in Singapore [23]. Although the Bayesian method yields reasonable uncertainty bounds of forecasts through the posterior distribution, a likelihood-based approach to improve our analytical understanding has yet to be considered. Accordingly, a simple likelihood-based model for forecasting that permits us to compute the prediction interval (the interval in which future observations will fall with a certain probability), is called for.

The aims of the present study are; (i) to develop a simple and practical approach to the real-time forecasting of an epidemic, and (ii) to apply the proposed method to a case study of pandemic influenza (H1N1-2009) in Japan. Here the empirical data for H1N1-2009 in Japan and technical problems of forecasting epidemics are described and a discrete time stochastic model that is analogous to an SIR epidemic model is derived. By imposing a branching process approximation to adhere to discrete time data, a simple method for computing the 95% prediction interval is proposed.

## Methods

### Description of the data

**Figure 1:**
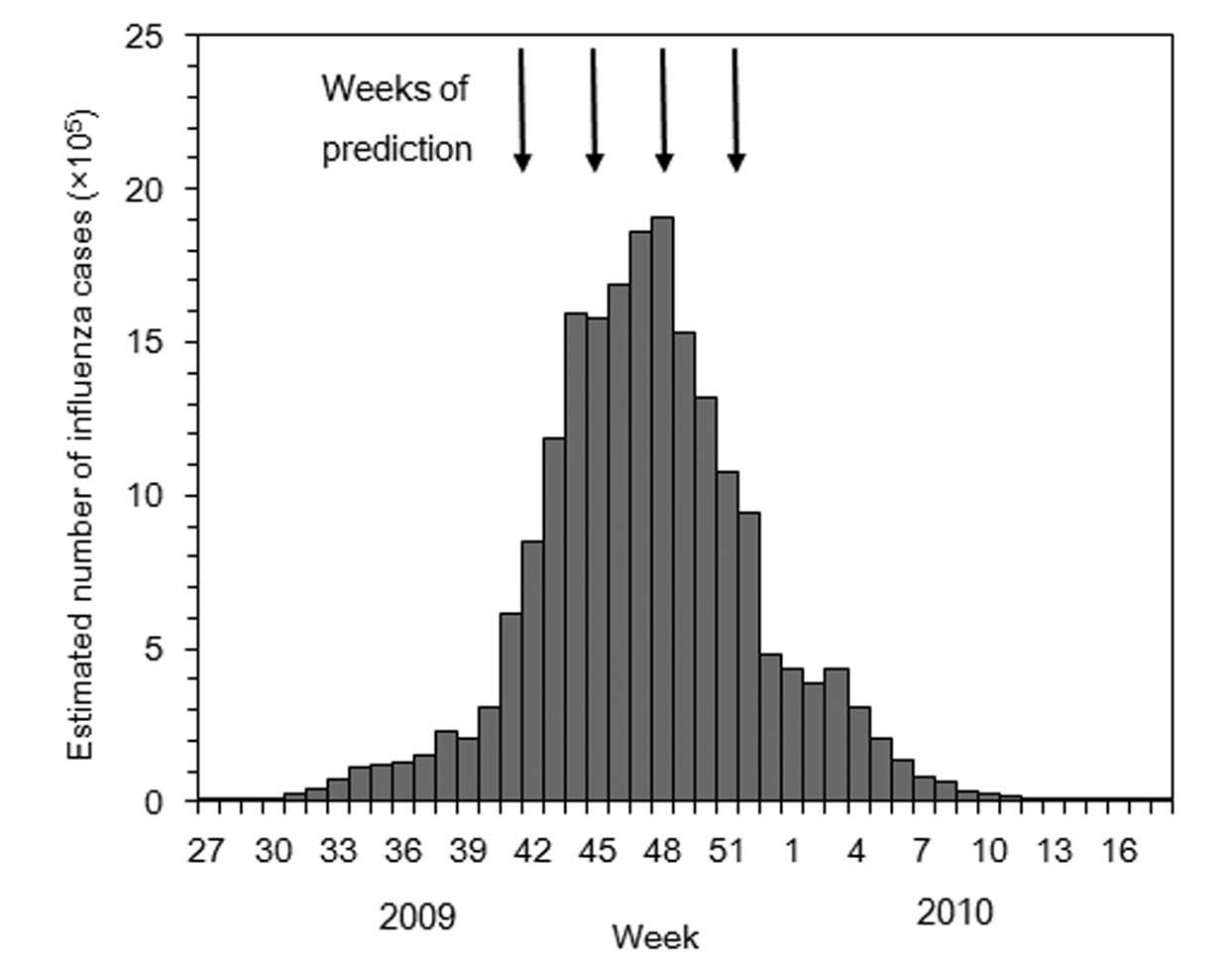
**Weekly incidence of influenza cases in Japan from 2009-10**. The vertical axis represents the estimated weekly number of cases based on a nationwide sentinel surveillance, covering the period from week 27 (the week ending on 5 July 2009) to week 18 (the week ending on 9 May 2010). The estimates, based on the notified number of cases from a total of 4800 randomly sampled sentinel hospitals, are extrapolated to the total number of medical facilities in Japan. The case represents all influenza-like illness cases that received medical attendance. During the period of interest, influenza A (H1N1-2009) dominated all influenza viruses that were isolated. The four arrows indicate the weeks (weeks 42, 45, 48 and 51 in 2009) that were used for the model predictions in the present study.

To clearly explain the motivation in carrying out this study, the empirical data of the pandemic (H1N1-2009) in Japan is first presented. Figure 1 shows the estimated weekly number of influenza cases based on national sentinel surveillance in Japan from week 27 in 2009 (the week ending 5 July) to week 18 in 2010 (the week ending 9 May). The estimates follow an extrapolation of the notified number of cases from a total of 4,800 randomly sampled sentinel hospitals to the total number of medical facilities in Japan. The notified cases represent patients who sought medical attendance and who met the following criteria, (a) acute course of illness (sudden onset), (b) fever higher than 38°C, (c) cough, sputum or breathlessness (symptoms of upper respiratory infection), and (d) general fatigue, or patients who were strongly suspected of having the disease and who undertook laboratory diagnosis (e.g. rapid diagnostic testing). Although the estimates of sentinel surveillance data have various epidemiological biases and errors, these issues have been ignored in the present study. For instance, by examining the information for test negative individuals, an unbiased estimate of true incidence of influenza (an estimate that excludes influenza-like illnesses due to other causes) could potentially be made [24]. However, no comprehensive data set is available and so the issue of misclassification is disregarded for now. During the period of interest, influenza A (H1N1-2009) substantially dominated all other isolated influenza viruses. The dynamics of confirmed cases during the very early epidemic phases have been reported elsewhere [25,26].

The aim of the present study is to forecast the future weekly incidence during the course of the epidemic. The four arrows in Figure 1 indicate the weeks of prediction (weeks 42, 45, 48 and 51 in 2009) that were selected to compare the validity of forecasting. These weeks were chosen for comparison because they are close to the peak and it is known that the forecasting of epidemics is of limited accuracy before the peak incidence is observed [18,22,23] and is likely to be greatly improved near the peak. The highest incidence was observed in week 48, so forecasts in weeks 42 and 45 represent those before the peak, in week 48 those at the peak and in week 51 those after the peak. To simplify the calculations that follow, the calendar weeks (week 27, 2009 to week 18, 2010) in which the data were collected have been set to match the actual weeks of the study (week 0 to 44).

Four major technical challenges for the real-time prediction should be noted. First, the observed epidemic curve represents only a single sample path (or a single stochastic realization) among all possible trajectories of the epidemic [27]. This implies that the model should account for stochastic variations in the data [28,29]. Second, because the virus is transmitted from host to host (human to human infection), an observation at time *t *depends on the previous series of observations up to time *t*-1 [30], reflecting the abovementioned dependent happening and statistically requiring conditional assessments. Third, any empirical data are reported and published at discrete time intervals, while, for the purpose of forecasting, ideal statistical data are continuous. The data in Figure 1 are based on weekly reporting which does not offer any information regarding the dynamics within each reporting interval. Fourth, the observed data usually involve reporting delays. Moreover, accounting for heterogeneity (spatial heterogeneity and social patterns of contact) and time-dependent epidemiological dynamics (seasonality of transmission, contact behaviors and public health interventions) is ideally required to give detailed insights into the epidemiological dynamics. Because the data in Figure 1 describe a single temporal distribution of the epidemic curve for an entire population of Japan, it does not have the information necessary to explicitly address these heterogeneities.

### Chain binomial model

Because the problems of delay and heterogeneity cannot be explicitly addressed without additional epidemiological information, data in Figure 1 are regarded as the weekly number of new infections (without any delay) generated by a homogeneously mixing population. It is also assumed that no intervention took place. These theoretical simplifications do not permit the interpretation of the model parameters explicitly in practical terms, and so the details of actual dynamics have, for now, been ignored. Rather, the focus is on the predictive performance of the simple model. The estimated parameters do retain practical interpretations for a hypothetical population in which the data generating process used for Figure 1 exactly follows the theoretical assumptions that are made.

To address the other three technical issues, in the present study a parsimonious discrete time stochastic model, which only accounts for intrinsic transmission dynamics using a small number of parameters, has been employed. To clearly describe the model-building process, the derivation of the model from the classical chain binomial model is shown. Let *S*_k _and *C*_k _represent the number of susceptible individuals and the weekly incidence (the number of new infections) in week *k*, respectively. Given *S*_k _and *C*_k_, the chain binomial model predicts *S*_k+1 _and *C*_k+1 _iteratively using binomial distributions [31]. The deterministic expression can be written as

(1) $$\begin{array}{l} E(C_{k+1})=S_{k}(1-\lambda_{k+1}),\\ E(S_{k+1})=S_{k}-E(C_{k+1}). \end{array}$$

where *λ*_k+1 _is the probability that susceptible individuals *S*_k _escape infection in week *k*+1 and E( ) represents the expected value; it should be noted that (1-*λ*_k+1_) can also be regarded as the discrete version of the force of infection in week *k*+1. Stochastically, the time series of incidence can be written as a chain of binomial random variables:

(2) $$C_{k+1}∼\mathrm{binomial}(S_{k},1-\lambda_{k+1}).$$

Equation (2) can more precisely be written as

(3) $$\mathrm{Pr}(C_{k+1}=x;S_{k}=s,\lambda_{k+1}=u)=\frac{s!}{x!(s-x)!}{\left(1-u\right)}^{x}u^{s-x}.$$

The Reed-Frost model is a special case of the chain binomial model that assumes $u=q^{C_{\text{k}}}$, where *q *is the probability of escaping infection from a single infected individual during week *k*+1 [32]. Although, in the present study, all the possible interpretations will not be discussed in detail, the case of *u *= *q *(i.e. *u *is independent of the number of infected individuals) is known to lead to the Greenwood model [31]. If *S*_0 _is the number of susceptible individuals at the beginning of the epidemic (week 0), then

(4) $$S_{k}=S_{0}-\sum_{h=1}^{k}C_{h},$$

and the probability of observing *C*_k _in week *k *can be conditioned on previous time series (up to week *k*-1) as:

(5) $$\begin{array}{l} \mathrm{Pr}(C_{0}\to C_{1}\to \cdots \to C_{k})\\ =\frac{S_{0}!}{C_{0}!\cdots C_{k}!S_{k}!}\prod_{i=0}^{k-1}{\left(1-\lambda_{i+1}\right)}^{C_{i+1}}\lambda_{i+1}^{S_{0}-\sum_{h=1}^{i+1}C_{h}}. \end{array}$$

Detailed properties of the Reed-Frost model are reviewed elsewhere [33]. Assuming that *λ*_k+1 _= exp(-*βC*_k_) and that the reporting interval is close to the infectious period of the disease of interest, the Reed-Frost model has been shown to be comparable to an SIR epidemic model with certain assumptions [31,34], and an extension of this type of Markov model has been applied to the real-time forecasting of influenza [23]. Despite its usefulness, the Reed-Frost model is not readily analyzed for large *S*_0 _(due to binomial arguments), and is mainly applicable to small populations. Although the issue of a large *S*_0 _has been addressed for computing the final size (i.e. the total number, or the proportion, of infections throughout the course of an epidemic) by means of the so-called Sellke construction [35,36], an approximate strategy is required for implementing real-time forecasting in a large population (see Barbour and Utev [37] for a detailed derivation of the approximation).

### An approximate branching process

As mentioned above, the chain binomial model can be related to the SIR epidemic model with some adjustment of the generation time [34] (the time interval between infection of a primary case and infection of a secondary case caused by the primary case [38]), although the crudely reported weekly data sometimes include a few generations of cases within each reporting interval. For instance, a contact tracing of H1N1-2009 in the Netherlands estimated the mean generation time as *T*_g _= 2.7 days [39], implying that weekly data can include more than two generations of influenza cases. Therefore, a different approach by imposing a linear argument to the dynamics within each reporting interval has been used.

**Figure 2:**
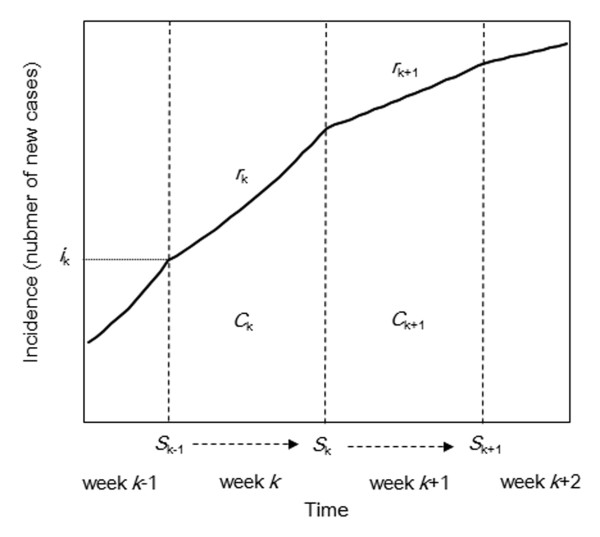
**Approximation of an epidemic curve**. The solid line represents the epidemic curve with assumed exponential growth within each reporting interval. The vertical dashed lines separate each reporting interval (week-wise). Growth rate in week *k *is assumed to be *r*_k_, and the area under the curve of week *k *(the cumulative incidence in each week) corresponds to the reported weekly incidence *C*_k_. Susceptible individuals in week *k*, *S*_k_, represent the number of susceptible individuals at the end of week *k*. The horizontal dotted line indicates the initial value of incidence, *i*_k _and represents the number of new cases at the beginning of week *k*.

Figure 2 illustrates the proposed approximation strategy. Because no information regarding the dynamics within each week is available, exponential growth in each week *k *with a growth rate *r*_k _is assumed. The area under the epidemic curve in week *k *(the cumulative incidence in week *k*) corresponds to the reported weekly incidence *C*_k_. Supposing that the initial value of incidence in week *k *is *i*_k_, then

(6) $$\begin{array}{c} E(C_{k})=\int_{0}^{\Delta t}i_{k}\exp(r_{k}s)ds\\ =\frac{i_{k}}{r_{k}}\left[\exp(r_{k}\Delta t)-1\right], \end{array}$$

and

(7) $$\begin{array}{l} E(C_{k+1})=\int_{0}^{\Delta t}i_{k}\exp(r_{k}\Delta t)\exp(r_{k+1}s)ds\\ =\frac{i_{k}\exp(r_{k}\Delta t)}{r_{k+1}}\left[\exp(r_{k+1}\Delta t)-1\right], \end{array}$$

where Δ*t *is the length of the reporting interval (7 days in this case). The deterministic iterative equation of *C*_k+1 _given *C*_k _can be written as ([21,40]):

(8) $$E(C_{k+1})=\frac{r_{k}\exp(r_{k}\Delta t)}{r_{k+1}}\frac{\exp(r_{k+1}\Delta t)-1}{\exp(r_{k}\Delta t)-1}C_{k}.$$

Susceptible individuals in week *k*, *S*_k_, represents the number of susceptible individuals at the end of week *k*. Because the growth of cases in each reporting interval is linearized,

(9) $$R_{k}=\frac{S_{k-1}}{S_{0}}R_{i},$$

where *R*_k _and *R*_i _are the instantaneous (effective) reproduction number in week *k *and the initial reproduction number (the average number of secondary cases generated by a single primary case in a susceptible population with size *S*_0_), respectively. We use the notation *R*_i_, instead of more commonly used *R*_0_, the basic reproduction number, because public health interventions took place during the 2009 pandemic, and the estimate of *R*_i _is greatly influenced by those interventions and other extrinsic factors [41,42]. Moreover, assuming that the generation time is a constant *T*_g _days (as is the case for the Reed-Frost model), then an estimator of the instantaneous reproduction number *R*_k _is ([43]):

(10) $$R_{k}=\exp(r_{k}T_{g}).$$

Equations (9) and (10) show that

(11) $$r_{k}=\frac{1}{T_{g}}\ln\frac{S_{k-1}R_{i}}{S_{0}}=\ln{\left(\frac{S_{k-1}R_{i}}{S_{0}}\right)}^{\frac{1}{T_{g}}}.$$

A more realistic distribution of the generation time could be adopted given a precise estimate of the variance, but in the present study only a constant generation time has been considered for simplicity. Replacing *r*_k _and *r*_k+1 _by *S*_0 _and *R*_i_, gives

(12) $$\begin{array}{l} E(C_{k+1})=\frac{{\left(\frac{S_{k-1}R_{i}}{S_{0}}\right)}^{\frac{\Delta t}{T_{g}}}\left[{\left(\frac{S_{k}R_{i}}{S_{0}}\right)}^{\frac{\Delta t}{T_{g}}}-1\right]\ln{\left(\frac{S_{k-1}R_{i}}{S_{0}}\right)}^{\frac{1}{T_{g}}}}{\left[{\left(\frac{S_{k-1}R_{i}}{S_{0}}\right)}^{\frac{\Delta t}{T_{g}}}-1\right]\ln{\left(\frac{S_{k}R_{i}}{S_{0}}\right)}^{\frac{1}{T_{g}}}}C_{k}\\ =A_{k}C_{k},\\ E(S_{k+1})=S_{k}-E(C_{k+1}). \end{array}$$

*A*_k _is the ratio of cumulative incidence in adjacent reporting intervals. The chain binomial model is analogous to a classical discrete time branching process model if it is assumed that the chain binomial model has a binomially distributed offspring distribution. Assuming a Poisson distribution for the observed counts of cases within each reporting interval for large *S*_0_, gives an alternative model:

(13) $$\mathrm{Pr}(C_{k+1}=x;C_{k},...,C_{0},S_{0},R_{i})=\frac{{\left(A_{k}C_{k}\right)}^{x}\exp(-A_{k}C_{k})}{x!}.$$

From equation (4), *A*_k _can be written as

(14) $$\begin{array}{l} A_{k}(S_{0},R_{i},C_{k},...,C_{0})\\ =\frac{{\left(\frac{R_{i}\left(S_{0}-\sum_{h=1}^{k-1}C_{h}\right)}{S_{0}}\right)}^{\frac{\Delta t}{T_{g}}}\left[{\left(\frac{R_{i}\left(S_{0}-\sum_{h=1}^{k}C_{h}\right)}{S_{0}}\right)}^{\frac{\Delta t}{T_{g}}}-1\right]\ln{\left(\frac{R_{i}\left(S_{0}-\sum_{h=1}^{k-1}C_{h}\right)}{S_{0}}\right)}^{\frac{1}{T_{g}}}}{\left[{\left(\frac{R_{i}\left(S_{0}-\sum_{h=1}^{k-1}C_{h}\right)}{S_{0}}\right)}^{\frac{\Delta t}{T_{g}}}-1\right]\ln{\left(\frac{R_{i}\left(S_{0}-\sum_{h=1}^{k}C_{h}\right)}{S_{0}}\right)}^{\frac{1}{T_{g}}}}. \end{array}$$

Assuming that *T*_g _is known (2.7 days), then the epidemic curve is governed by only two parameters, *S*_0 _and *R*_i_. Thus, an SIR model with a constant generation time has been simplified to a branching process model that explicitly accounts for the practical interpretation of the observed weekly cumulative incidence *C*_k_.

### Statistical estimation and computation of the uncertainty bounds

The statistical estimation of *S*_0 _and *R*_i_, given observed incidence data up to week *K*, is straightforward. Given the time series of weekly incidence *C*_0_, ..., *C*_K_, the conditional likelihood function to estimate *S*_0 _and *R*_i _is

(15) $$L(S_{0},R_{i})=\prod_{h=1}^{K}\frac{{\left(E(C_{k})\right)}^{C_{k}}\exp(-E(C_{k}))}{C_{k}!}.$$

where E(*C*_k_) = *A*_k-1_(*S*_0_, *R*_i_, *C*_k-1_, ..,*C*_0_)*C*_k-1_. That is, the likelihood in week *k *is conditioned on the previous week *k*-1; a process that is commonly adopted to address stochastic dependence structures in any relevant nonlinear models [44]. The maximum likelihood estimates are obtained by minimizing the negative logarithm of equation (15). The 95% confidence intervals (CIs) of the parameters are derived from profile likelihoods. Using the maximum likelihood estimates based on the data from week 0 to *K*, and assuming that we have an unbiased maximum likelihood estimate of *R*_i_, the final size, (the proportion of infections by the end of an epidemic), *z*, is computed by iteratively solving the following final size equation that is derived from a continuous SIR model [45]:

(16) $$1-z=\exp(-z{\hat{R}}_{i}).$$

The total number of cases throughout the course of an epidemic, *Q*, is then given by

(17) $$\hat{Q}=\hat{z}{\hat{S}}_{0}.$$

The 95% CI of the final size *z *is approximately computed using the Wald method and employing the approximate standard error of *z *[28]:

(18) $$\begin{array}{l} \left(z_{L},z_{U}\right)\\ =\left(\hat{z}-1.96\sqrt{\frac{(1-\hat{z}){\hat{z}}^{3}}{{\hat{S}}_{0}{\left(\hat{z}+(1-\hat{z})\ln(1-\hat{z})\right)}^{2}}},\hat{z}+1.96\sqrt{\frac{(1-\hat{z}){\hat{z}}^{3}}{{\hat{S}}_{0}{\left(\hat{z}+(1-\hat{z})\ln(1-\hat{z})\right)}^{2}}}\right), \end{array}$$

which is valid only in the case of a constant generation time.

The computation of prediction intervals can employ chains of the conditional offspring distributions (equation (13)). First, to address parameter uncertainty, *S*_0 _and *R*_i _are randomly sampled 1,000 times from uniform distributions ranging from the lower to upper 95% CIs [46]. Second, from the 1,000 combinations of the two parameters, minimum and maximum values of the results from the following calculations are chosen as the lower and upper prediction intervals, respectively. For each combination of the parameters, the upper and lower 1-2*ε *limits of *C*_K+1 _in week *K*+1 are the largest *C*_U _and the smallest *C*_L _such that

(19) $$\varepsilon \leq \mathrm{Pr}(C_{U})=\sum_{r=C_{U}}^{\infty }\mathrm{Pr}(C_{K+1}=r;C_{K},...,C_{0},{\hat{S}}_{0},{\hat{R}}_{i}),$$

and

(20) $$\varepsilon \leq \mathrm{Pr}(C_{L})=\sum_{r=0}^{C_{L}}\mathrm{Pr}(C_{K+1}=r;C_{K},...,C_{0},{\hat{S}}_{0},{\hat{R}}_{i}).$$

For week *K*+1 only, the sums can be found by a computationally efficient method that uses the incomplete gamma function. In week *K*+2, the lower and upper prediction intervals are computed as

(21) $$\begin{array}{l} \varepsilon \leq \mathrm{Pr}(C_{U})=\sum_{r=C_{U}}^{\infty }\mathrm{Pr}(C_{K+2}=r,C_{K+1}=s;C_{K},...,C_{0},{\hat{S}}_{0},{\hat{R}}_{i})\\ =\sum_{r=C_{U}}^{\infty }\sum_{s=0}^{\infty }\mathrm{Pr}(C_{K+2}=r;s,{\hat{S}}_{0},{\hat{R}}_{i})\mathrm{Pr}(C_{K+1}=s;C_{K},...,C_{0},{\hat{S}}_{0},{\hat{R}}_{i}), \end{array}$$

and

(22) $$\begin{array}{l} \varepsilon \leq \mathrm{Pr}(C_{L})=\sum_{r=0}^{C_{L}}\mathrm{Pr}(C_{K+2}=r,C_{K+1}=s;C_{K},...,C_{0},{\hat{S}}_{0},{\hat{R}}_{i})\\ =\sum_{r=0}^{C_{L}}\sum_{s=0}^{\infty }\mathrm{Pr}(C_{K+2}=r;s,{\hat{S}}_{0},{\hat{R}}_{i})\mathrm{Pr}(C_{K+1}=s;C_{K},...,C_{0},{\hat{S}}_{0},{\hat{R}}_{i}), \end{array}$$

because all possible chains for both weeks *K*+1 and *K*+2 have to be considered. The sums have to be calculated directly. Similarly, for a later week *K*+*m*, the sums of all possible chains in weeks *K*+1, *K*+2, ..., *K*+*m*-1 have to be computed. Although finding *C*_U _and *C*_L _for later chains requires a computer programming code, the chain Poisson model still remains computationally very simple. Alternatively, a negative binomially distributed offspring distribution [47] in which a dispersion parameter has to be jointly estimated could be used.

Using the simple model described above, real-time forecasting of influenza (H1N1-2009) was visually evaluated at weeks *K *= 15, 18, 21 and 24. By comparing the parameter estimates against those derived from an entire epidemic curve (using estimates based on the data from weeks 0 to 44) the accuracy of the real-time estimation of the parameters was assessed. The mean generation time *T*_g _was fixed at 2.7 days. In addition to a visual assessment of the forecasts, the mean absolute error (MAE) was computed continuously for the weeks of prediction from weeks 5 to 35, and used to measure the closeness of forecasts (E(*C*_k_)) to the observed data (*x*_k_), i.e.,

(23) $$\mathrm{MAE}=\frac{1}{n}\sum_{i=1}^{n}\left|E(C_{i})-x_{i}\right|.$$

where *n *is the number of weeks of observation involving conditional expectation or prediction (*n *= 44 in the case study). MAE was chosen to measure the validity of forecasting, because (i) the scale does not directly influence the assessment of the predictions as a whole nor does it affect the comparative examination by week of prediction and (ii) the comparison is made against a single observed time series data set [48].

## Results

### Parameter estimates

Table 1 summarizes parameter estimates obtained using the weekly incidence data. At different weeks of prediction, maximum likelihood estimates of *R*_i _ranged from 1.14 to 1.18 which was broadly consistent with the estimate based on the entire epidemic curve (*R*_i _=1.13). The CIs overlapped with the CI in week 44, although the 95% CI based on week 15 was broad, ranging from 0.88 to 1.40. It should be noted that because the epidemic was affected by seasonality, public health interventions and heterogeneous mixing, the estimated *R*_i _is not useful as a practical measure to be considered for disease control. *R*_i _may not, for example, be useful when considering the required coverage of vaccination for disease containment whereas the basic reproduction number, *R*_0 _could be used. Rather, the estimated *R*_i _represents the transmission potential for an epidemic curve generated by a hypothetical homogeneously mixing population. Therefore, if the model fully captures the underlying epidemiological dynamics, the results would indicate that the transmission potential could be accurately estimated using the proposed method.

**Table 1:** Estimates of parameters for the proposed model using weekly incidence data of influenza (H1N1-2009) in Japan

Week of prediction*	Initial reproduction number	Initially susceptible individuals (×10^5^)	Total number of cases (×10^5^)^†^	MAE^‡^
15	1.14 (0.88, 1.40)	113083 (0, 256710827)	26778 (25826, 27749)	663
18	1.18 (1.10, 1.28)	391 (218, 741)	573 (66, 1637)	1.9
21	1.15 (1.07, 1.21)	754 (0, 2225)	183 (105, 261)	0.6
24	1.15 (1.09, 1.20)	716 (540, 1104)	175 (100, 251)	0.6
44	1.13 (1.09, 1.18)	834 (664, 1149)	188 (101, 274)	0.5

The values in parenthesis are the 95% CIs. The 95% CIs for the initial reproduction number and initial number of susceptible individuals were derived from profile likelihood, while those for the total number of cases were computed using an approximate standard error of the final epidemic size. *Week by which the data were available. Using the data from week 0 to the specified week, two parameters (*R*_i _and *S*_0_) were estimated and forecasts for later weeks were made. Week 44 corresponds to the end of the observation period, and the parameter estimates are based on the conditional fitting procedure using the data of the entire epidemic curve from weeks 0 to 44. ^†^Estimated total number of cases from week 0 to 44, including conditionally expected values from week 0 to the week of prediction (*t*) and forecasting from *t*+1 to 44. ^‡^MAE, mean absolute error; an average of absolute differences between observed and predicted values, representing a measurement of forecast error throughout the course of the epidemic.

The estimate of *S*_0 _differed greatly depending upon the weeks of prediction. At week 15, *S*_0 _was overestimated to the extent that it exceeded the actual population of Japan (approximately 1200×10^5^). Although an advantage of the proposed stochastic model is its potential to estimate *S*_0 _from incidence data, the estimates of *S*_0 _before the epidemic peak appeared to be inaccurate. Based on the entire epidemic curve, *S*_0 _was estimated to be 834×10^5^, indicating that 69.5% of the Japanese population was initially susceptible. Given that the estimate agrees well with the result of serological surveillance [49], *S*_0 _for the entire epidemic curve may be validly quantified even without the population data. Despite slight underestimations, the estimates of *S*_0 _at and after the epidemic peak are close to the estimate based on week 44 with overlapping CIs.

### Prediction

**Figure 3:**
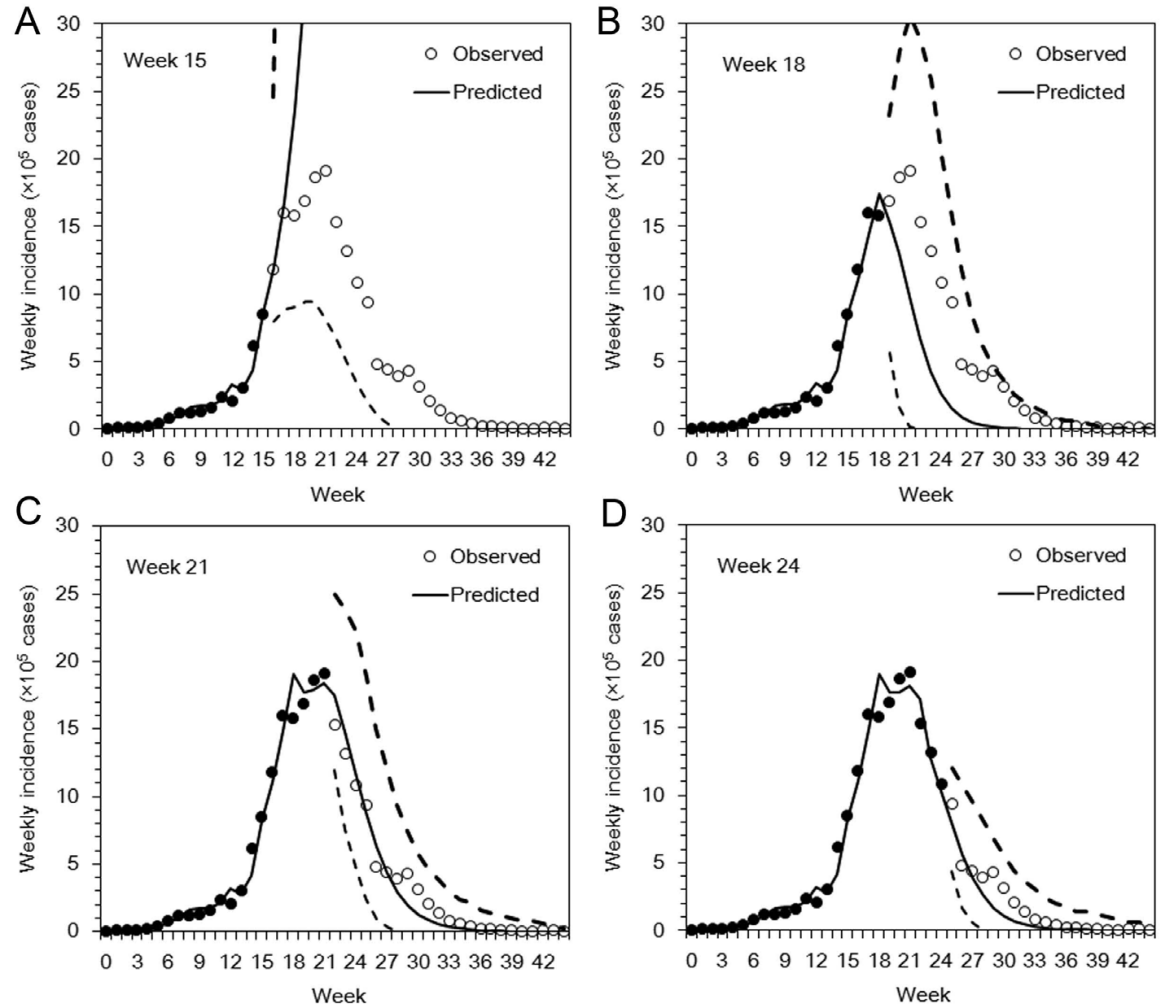
**Assessment of influenza forecasts**. The observed weekly incidence of influenza cases (circles) are compared against the predicted number of cases (lines). Filled circles represent the observed data that were used for prediction, and unfilled circles represent the observed data that was predicted using the proposed method. The unbroken line represents the expected prediction made using maximum likelihood estimates of the model parameters, and the dashed lines show the lower and upper 95% prediction intervals from the proposed uncertainty analysis. Panels A, B, C and D show the impact the different weeks (at weeks 15, 18, 21 and 24, respectively) on predictions of the future course of the epidemic. During the period of observation the unbroken line represents the conditionally expected values, while during the period of forecasting the line represents the conditionally predicted values. Week 0 on the horizontal axis corresponds to week 27 (week ending on 5 July 2009).

The observed and predicted weekly incidence are plotted and displayed in Figure 3. At week 15 the forecast failed to predict the epidemic with very broad 95% prediction intervals because of the overestimation of *S*_0 _mentioned above (Figure 3A). Using the maximum likelihood estimates of parameters, the peak weekly incidence was predicted to range from 2298×10^5 ^to 2834×10^5^. As seen in previous studies [18,22,23], the model prediction is sensitive to variations in the growth rate of incidence before the epidemic peak appears. In other words, the validity of forecasts before the epidemic peaks largely depends on obtaining good parameter estimates, and addressing this limitation is difficult if forecasts are based only on crudely reported weekly incidence data. At week 18, shortly before the peak, the prediction captured the shape of epidemic curve qualitatively, but the expected values of the forecast underestimated the weekly incidence (Figure 3B). Using maximum likelihood estimates, the estimated peak weekly incidence ranged from 12×10^5 ^to 19×10^5^. At the peak and after the peak, the prediction dramatically improved. All the observed incidences at weeks 21 and 24 were within the 95% prediction intervals (Figure 3C and 3D).

**Figure 4:**
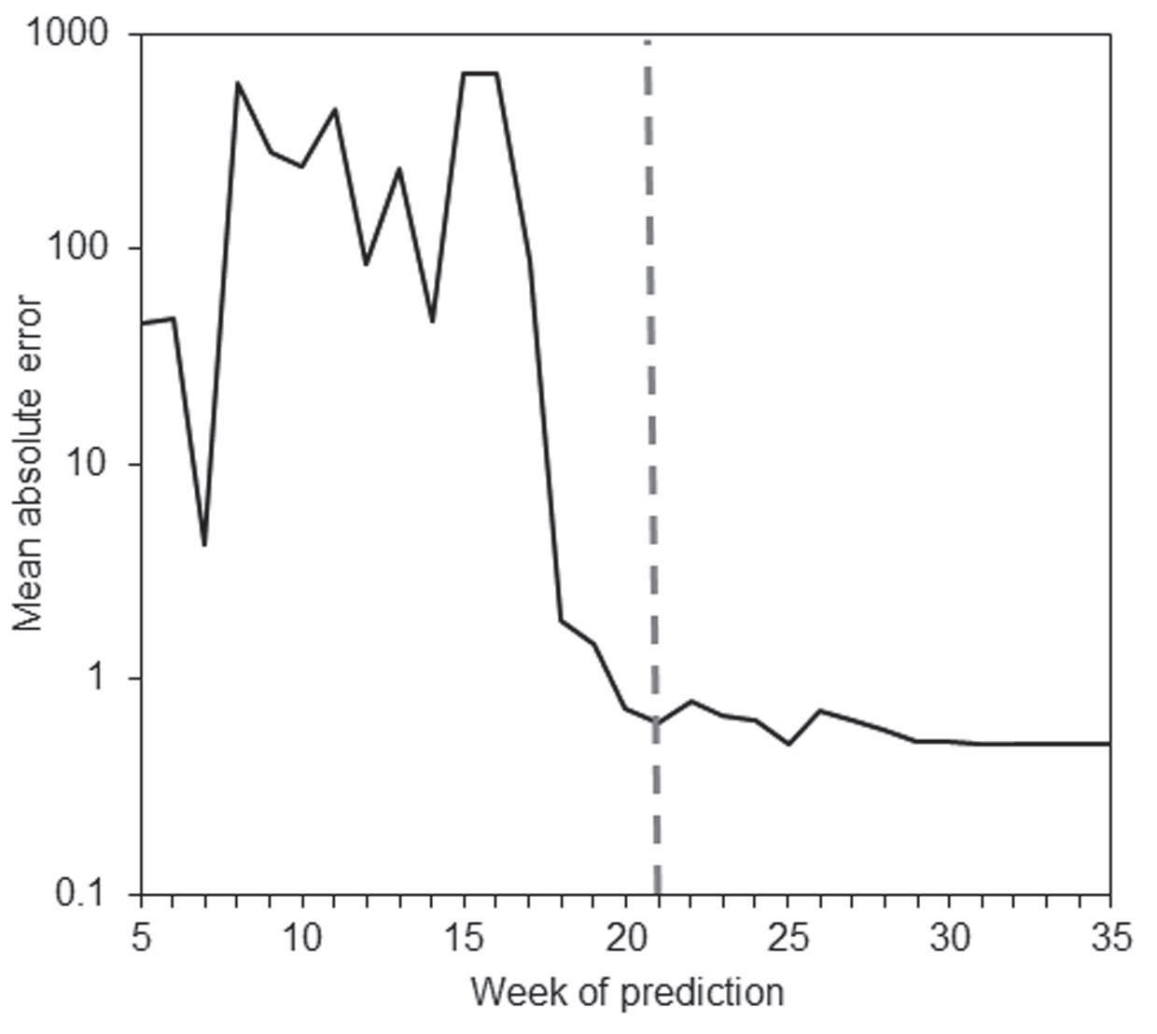
**Mean absolute error by week of prediction**. The vertical axis shows an average of absolute differences between observed and predicted values that represent the forecast error throughout the course of the epidemic. It should be noted that the vertical axis is in logarithmic scale. The dashed vertical line indicates the week at which the largest incidence (the peak) was observed (week 21). The horizontal axis represents the week of prediction.

Despite accurate estimates of *R*_i, _because of the large variation in the estimates of *S*_0_, the predicted final size varied greatly with the week of prediction (Table 1). The observed total number of cases was 203×10^5 ^and at week 44 the model slightly underestimated the final size perhaps because of the approximate linear modeling approach to the epidemic curve, however, the observed value was within the 95% CI. Although the prediction at week 18 underestimated the final size, the predicted final size at weeks 21 and 24 was included within the 95% CIs. In addition to the data given in Table 1, Figure 4 shows continuously evaluated MAE values for the weeks of prediction from weeks 5 to 35. The error fluctuated and was extremely large before the peak of the epidemic curve. At and after the peak the error was greatly reduced, reflecting the accuracies of forecasts mentioned above. In the present case study, an abrupt decline in MAE was seen in week 18, three weeks before observing the peak incidence (Figure 4).

Even in week 15, assuming that *S*_0 _is known (set as 834×10^5 ^persons based on week 44), the epidemic curve described by *R*_i _alone qualitatively captured the observed epidemic curve (figure not shown). *R*_i _was estimated to be 1.16 (95% CI: 1.08, 1.23) and the MAE was reduced to 1.26, indicating that early forecasting is sensitive to variations in *S*_0 _which is influenced by variations in the growth rate. When a constraint for the upper boundary of *S*_0 _using the entire population size for Japan (say, 1200×10^5^) was imposed for the prediction at week 15, *R*_i _and MAE were, estimated at 1.15 and 2.96, respectively (the original MAE at week 15 was 663 as shown in Table 1). Clearly, the validity of the prediction was greatly improved by using a constraint on the population size. Nevertheless, it should be noted that the use of a constrained *S*_0 _imposes the arbitrary assumption that the entire population was initially susceptible and was fully involved in the transmission dynamics.

## Discussion

The present study has proposed a method for real-time forecasting based on crudely reported weekly incidence data, accounting for demographic stochasticity and conditional measurement and employing a simple discrete time stochastic model. The proposed model was constructed using a branching process approximation of a chain binomial model. In particular, realizing that the weekly incidence data of influenza *C*_k _is less interpretable than the incidence data of other diseases with longer generation times (e.g. measles), the iterative model was parameterized by assuming exponential growth of cases within each reporting interval. Consequently, the parsimonious model resulted in a novel, yet fully tractable form. Although the proposed stochastic model is analogous to models with a series of chains, it can incorporate a more realistic distribution of the generation time and, given more detailed epidemiological information, has a broad range of extensions. Moreover, the chains of Poisson offspring distributions enable the computation of the 95% prediction intervals. It is known that a non-linear model does not allow simple computation of the prediction interval [50] and, although a more formal approach to computing the prediction interval should ideally account for future observations more explicitly (and to be strict, the prediction interval of the present study may better be referred to as the forecast region), the proposed approach is not very computationally demanding.

The biggest advantage of the proposed model is its potential to describe and predict the epidemic curve with interpretable parameters *S*_0 _and *R*_i _under a homogeneous mixing assumption. In addition, the parameterization produces estimates that can be exploited to compute the final epidemic size. Nevertheless, as was observed in other attempts at real-time forecasting [18,22,23], the forecast appears to be very vulnerable to the timing of forecasting, especially during the early growth phase of an epidemic. Indeed, Figure 4 has captured the difficulty of early forecasting in terms of the MAE. Although, even at week 15, the qualitative behavior of forecasts is greatly improved by fixing *S*_0 _or by imposing constraints for *S*_0 _(and leaving only *R*_i _as a free parameter), the advantage of the proposed model is in its ability to estimate *S*_0 _explicitly. Indeed, in practical settings it may be best to assume that *S*_0 _is an unobserved variable. It should be noted that the results also imply that serological surveillance before and during an epidemic may be a great help in improving the forecasts [21].

Despite the omission of heterogeneity, when more precise data in time and structure becomes available, it can readily be incorporated into the proposed model. For example, the model can potentially be extended for age-dependent and spatially structured data like that used to compute the final epidemic size in a multi-host population [51]. Such an extension could potentially begin to address the difficulty of real-time forecasting in the presence of a multimodal epidemic curve. That is, given that a few peaks in a single temporal distribution resulted from multiple epidemic curves in different spatial units [49], the spatial extension could capture different epidemic waves in different geographic areas [52]. Another important future task is to allow the model to fully adhere to the data generating process. If the reporting delay and any time-dependent epidemiological information (e.g. data that are likely to inform a time-dependent covariate of the risk of infection) are known, the proposed model could potentially incorporate those aspects in the model-building strategy. The impact of such complexities on forecasting should be explored when the required information becomes available as part of the surveillance.

As was shown through the likelihood-based approach, the present study has demonstrated that real-time forecasting can rest on a simple discrete time stochastic model and has shown that the uncertainty bounds can reasonably be computed using the conditional offspring distributions. Despite the simplicity, the present study successfully offers a sound modeling strategy and a methodological avenue to implement real-time forecasting of an epidemic in the midst of its course.

## Conclusions

Because real-time forecasting of epidemics has been understudied, in the present study a discrete time stochastic model, accounting for demographic stochasticity and conditional measurement was developed. The model permitted us to derive the uncertainty bounds using chains of conditional offspring distributions. The proposed method was applied to the weekly incidence of pandemic influenza (H1N1-2009) in Japan. The validity of forecasts made before the epidemic peak appeared, largely to depend on obtaining good parameter estimates, and the forecasts of both weekly incidence and final epidemic size greatly improved at and after the peak with all the observed data points falling within the uncertainty bounds. Because the structure of the proposed model is simple, it has the potential to additionally account for heterogeneity, time-dependent transmission dynamics and epidemiological details when that information becomes available as part of the data generating process.

## Competing interests

The author declares that they have no competing interests.

## Authors' contributions

HN developed the methodological ideas, implemented the mathematical and statistical analyses, and drafted the manuscript. The author read and approved the final manuscript.
